# Supracricoid laryngectomy: the impact of senescence on swallowing safety

**DOI:** 10.31744/einstein_journal/2021AO5715

**Published:** 2021-04-28

**Authors:** Andressa Silva de Freitas, Guilherme Maia Zica, Ana Catarina Alves e Silva, Fernando Luiz Dias, Emilson Queiroz Freitas, Izabella Costa Santos

**Affiliations:** 1 Instituto Nacional de Câncer José Alencar Gomes da Silva Rio de JaneiroRJ Brazil Instituto Nacional de Câncer José Alencar Gomes da Silva - INCA, Rio de Janeiro, RJ, Brazil.; 2 Fundação Oswaldo Cruz Rio de JaneiroRJ Brazil Fundação Oswaldo Cruz, Rio de Janeiro, RJ, Brazil.

**Keywords:** Laryngectomy, Rehabilitation, Deglutition, Aging, Deglutition disorders

## Abstract

**Objective:**

To investigate the association between aging and the functional aspects of swallowing (laryngeal penetration and laryngotracheal aspiration) in individuals who underwent supracricoid laryngectomy in the late period and without complaints.

**Methods:**

A total of 70 patients, 56 (80%) aged >60 years and 14 (20%) <60 years, under outpatient follow-up, after cancer treatment and with no complaints of swallowing, performed functional evaluation using the swallowing videofluoroscopy. Image classification was performed using the penetration-aspiration scale developed by Rosenbek. The *χ*^2^ test and logistic regression were applied to associate the age categories to the outcomes (penetration and aspiration).

**Results:**

Patients aged over 60 years had a higher prevalence of penetration (24.29%) and aspiration (48.57%) than patients aged under 60 years. In this sample, aspiration was associated with age. Patients aged over 60 years were more likely to present penetration (27% more) during swallowing than patients under 60 years. Patients aged over 60 years had an approximately four-fold greater probability of laryngotracheal aspiration than patients aged under 60 years.

**Conclusion:**

In patients without complaints of swallowing in the late postoperative period of supracricoid laryngectomy, there is a greater probability of laryngotracheal aspiration in elderly aged over 60 years than in individuals under 60 years.

## INTRODUCTION

Aging is a natural process that results in gradual and inevitable age-related changes. Besides triggering organic and functional wear and tear, it promotes changes in emotional, social, and cultural aspects.^(^^1^^)^ Normal aging is linked to the individual’s ability to adapt to the rigors and aggressions of the environment. Thus, each subject ages in their own way, depending on several variables, such as sex, origin, family environment, work, occupations, and experiences lived.^(^^1^^–^^3^^)^ For the World Health Organization (WHO), the age limit between the adult and the elderly is 65 years in developed nations and 60 years in emerging countries.^(^^4^^)^

Exposure to stress or smoking, lack of exercise, or inadequate nutrition are factors that contribute to determine the quality of the individual’s aging.^(^^2^^)^ Senescence and disease cannot be treated as closely dependent or interrelated factors, but it is possible to understand that the elderly person is more vulnerable when ill.^(^^3^^)^

Dysphagia is a dysfunction of swallowing, defined by the difficulty or inability to move effectively the food ingested from the mouth to the stomach. This disorder can affect up to 13% of population aged 65 and over, and 51% of institutionalized elderly individuals.^(^^5^^)^ Swallowing can be impacted by increased age, accumulation of comorbidities, and hospitalization. There are several diseases with the potential to trigger dysphagia, such as stroke, and head and neck tumors. In these groups, studies suggest that their prevalence increases with survival of individuals, which may mean that the changes in aging contribute to the aggravation of dysphagia.^(^^6^^,^^7^^)^

Supracricoid laryngectomy (SCL) is a partial horizontal surgical procedure indicated for T1b to T4a tumors of the glottic and supraglottic region, and its main advantages are partial preservation of laryngeal functions and absence of a permanent tracheostomy.^(^^8^^)^ Its technique consists of removing the pedicle from the epiglottis, thyroid cartilage, laryngeal ventricles, vocal folds, vestibular folds, and paraglottic space. One or both cricoarytenoid units, the epiglottis and the cricoid, remain. Its surgical reconstruction occurs, among variations, through the cricohyoidoepiglotopexy (CHEP), in which a suture is made between the cricoid cartilage, the epiglottis, and the hyoid bone, raising the laryngeal complex to the level of the hyoid bone.^(^^8^^,^^9^^)^ Supracricoid laryngectomy promotes changes in the biodynamics of swallowing due to the loss of important lower airway protection structures.^(^^10^^,^^11^^)^

Pharyngeal muscle atrophy has been identified as a natural biological process of aging, in which an increase in pharyngeal area and volume is observed in healthy individuals. It is known that healthy elderly people with larger pharyngeal cavities present with worsening of pharyngeal constriction and, consequently, more residues in pharyngeal recesses and vallecula.^(^^5^^)^ Natural aging promotes calcification of laryngeal cartilages, less muscle mobility, and weak healing/vascularization in the postoperative period, which is negatively related to surgical issues.^(^^7^^,^^9^^,^^11^^)^ It is believed that aging can enhance the negative oncological results of laryngectomies by different factors. This aspect can gradually compromise the swallowing process, due to the sum of the presbyphagia and the anatomophysiological rearrangement of the neoglottis.

## OBJECTIVE

To investigate the association between aging and the functional aspects of swallowing in individuals submitted to supracricoid laryngectomy in the late period.

## METHODS

This is a cross-sectional study conducted at the Head & Neck Cancer Surgery Department of the *Instituto Nacional de Câncer do Brasil* (INCA), Rio de Janeiro (RJ), Brazil. Approval for this study was obtained from the Teaching and Research Committee of the organization, under opinion 3.892.889, CAAE: 26331314.2.0000.5274.

The different ages for defining the elderly according to the WHO exist because of lower survival rates and issues associated with public health.^(^^4^^,^^12^^)^ Age, in this study, was considered at the time of evaluation, not during surgery. Seventy patients, 56 (80%) >60 years and 14 (20%) <60 years, in outpatient follow-up after oncologic treatment and without swallowing complaints, underwent functional evaluations by means of videofluoroscopy swallowing study (VFSS). The images were classified using the Penetration and Aspiration Scale developed by Rosenbek et al., in 1996, and used as a parameter to analyze the presence and absence of laryngeal penetration and laryngotracheal aspiration.^(^^13^^)^

As inclusion criteria, patients had to be older than 18 years, had been surgically treated by the technique described by Majer et al.,^(^^8^^)^ and improved by Laccourreye et al.,^(^^9^^)^ with no active disease at the time, and with at least 6 months of oncologic treatment, with no local recurrence or distant metastases. The patients presented with no complaint of swallowing, discharge from the speech therapy service, and no tracheostomy or feeding tube. Adjuvant radiation therapy as part of the treatment protocol was not an exclusion criterion. Patients with cognitive and/or linguistic impairment were excluded, as were those who underwent other types of surgical intervention in the head and neck region before or after SCL with CHEP.

The videofluoroscopic studies were performed at the Radiology Department of INCA, according to the Logemann-based^(^^14^^)^ protocol, and adapted at the Head and Neck Interdisciplinary Laboratory (LICEP - *Laboratório Interdisciplinar de Cabeça e Pescoço*). A remote-controlled Siemens Axiom Iconos MD X-ray machine, serial number 13020, was used. All video segments were recorded on side view, with an image capture rate of 30 frames per second, and archived in Picture Archiving and Communication Systems (PACS), allowing the storage of documents for further review and analysis.

The consistency preparation protocol was the contrast medium offered in a glass, using dilutions of 100% barium sulfate (BS) (Bariogel^®^), mineral water, and food thickener Resource^®^ ThickenUp Clear. Four consistencies were offered: liquid 5mL (2.5mL of water + 2.5mL of BS), 10mL (5mL of water + 5mL of BS), and 20mL (10mL of water + 10mL of BS); semiliquid 5mL of BS, 10mL of BS, and 20mL of BS; pasty food 5mL (5mL of BS + 1.2g of thickener), 10mL (10mL of BS + 2.4g of thickener), and 20mL (20mL of BS + 3.6g of thickener); solid (cookie moistened in BS). During the test, all subjects were positioned in a side view, as close as possible to the table and intensifier, to avoid distortions in the fluoroscopic image.

This is a study with descriptive analysis and simple statistical regression. The *χ^2^* test and logistic regression were applied to associate the age categories to the outcomes (penetration and aspiration) using the R-3.5.3. software.

## RESULTS

We evaluated 70 patients, predominantly elderly, aged over 60 years (80%), male (98.57%), married (75.71%), and with a low level of schooling (57.14%). More than 80% of individuals were smokers, and at least 60% reported alcoholism at the time of diagnosis. Adding the single, divorced, and widowed categories, we obtained 24.28% (n=17) who did not report having a companion at the time of collection. As to staging, stages I and II were observed in 38.57% and 32.86% of individuals, respectively (Table 1).

**Table 1:** Distribution of information on habits, clinical variables, and sociodemographic data

Variables		
Sex		
	Male	69 (98.6)
	Female	1 (1.4)
Age in July 2017, in years		
	<60	14 (20)
	≥60	56 (80)
Marital status		
	Married	53 (75.7)
	Single	8 (11.4)
	Divorced	3 (4.3)
	Widowed	6 (8.6)
Schooling level		
	Illiterate	2 (2.9)
	Primary School	48 (54.3)
	Middle School	25 (35.7)
	College	5 (7.1)
Smoker upon diagnosis		57 (81.4)
Alcohol user upon diagnosis		46 (65.7)
Clinical staging		
	I	19 (27.1)
	II	27 (38.6)
	III	23 (32.9)
	IV	1 (1.4)
Nasogastric tube, days		
	≤45	38 (54.3)
	46-89	20 (28.6)
	>90	12 (17.1)
Tracheostomy, days		
	≤45	46 (65.7)
	46-89	12 (17.1)
	>90	12 (17.1)
Radiation therapy		22 (31.9)
Arytenoids preserved		
	1	32 (47.7)
	2	38 (54.3)

Results expressed by n (%).

Nasogastric tube and tracheostomy removals occurred up to 45 days in 54.28% and 65.71%, respectively. Radiation therapy was performed in 31.9% of patients (Table 1).

Patients aged over 60 years had a higher prevalence of penetration (24.29%) and aspiration (48.57%) than those younger than 60 years. In this sample, aspiration was associated with age (Table 2).

**Table 2:** Distribution of the group by age, according to prevalence of penetration and laryngeal aspiration

Age, years	Penetration			Aspiration		
	Yes	No	p value	Yes	No	p value
<60	5 (7.14)	9 (12.86)	0.787	12 (17.14)	2 (2.86)	
≥60	17 (24.29)	39 (55.71)		34 (48.57)	22 (31.43)	0.063

Results expressed as n (%).

Patients older than 60 years had a 27% greater probability of penetration during swallowing than those younger than 60 years. They were approximately four times more likely to present laryngotracheal aspiration than patients under 60 years (Table 3).

**Table 3:** Simple group regression by age, according to the prevalence of penetration and laryngeal aspiration

Age, years	Penetration			Aspiration		
	OR	95%CI	p value	OR	95%CI	p value
<60	1			1		
≥60	1.27	0.35-4.28	0.700	3.88	0.94-26.51	0.094

OR: odds ratio; 95%CI: 95% confidence interval.

## DISCUSSION

The motivation for this study was based on the need to understand the correlation between presbyphagia and safety and efficacy of swallowing in patients undergoing SCL CHEP. The technique in question aims to partially maintain the swallowing and phonation functions as an alternative to treatments with greater potential to cause functional modifications, such as exclusive radiation therapy and total laryngectomy, in the case of advanced tumors.^(^^8^^,^^9^^,^^15^^)^ Data found in this study suggest a greater probability of laryngotracheal aspiration in individuals aged over 60 years submitted to SCL CHEP when compared to those younger than 60 years submitted to the same procedure, both without complaints of swallowing or oral route limitations.

Supracricoid laryngectomy CHEP is a traditionally prescribed surgical procedure for the treatment of intermediate and advanced stage tumors, which justifies the prevalence of individuals with clinical staging two and three (71.5%).^(^^9^^,^^11^^,^^16^^–^^18^^)^ The procedure has proven to be a viable alternative to total laryngectomy, with partial preservation of laryngeal functions in all individuals.

Tracheal aspiration is a known chronic clinical outcome of SCL, with approximately 40% incidence in the postoperative period, even in individuals without complaints.^(^^17^^–^^19^^)^ *Deficits* in glottic closure and airway protection, due to the removal of approximately 70% of organ, and changes in sensitivity in the neolarynx may partially justify such findings.^(^^19^^)^

Patients aged over 60 years had a higher prevalence of penetration and aspiration during the objective analysis of swallowing. Several factors in presbyphagia may influence the functional outcome of elderly individuals, such as loss of weight and muscle mass, reduced tissue elasticity, changes in the cervical spine, reduced saliva production, impaired dental status and oral health, reduced oral and pharyngeal sensitivity, reduced olfactory and gustatory function, and reduced brain compensatory capacity.^(^^5^^,^^6^^,^^12^^,^^20^^)^ Evidently, all these aspects increase the susceptibility to dysphagia and overlap with the surgical complications of oncologic treatment.

It is believed that the changes arising from aging can affect all phases of swallowing, regardless of the association with other comorbidities. Prolonged oral phase, reduced tongue pressure, delayed swallowing reflex, delayed laryngeal closure, decreased swallowing volume, and increased residual and penetration rate are described as typical changes of older people.^(^^20^^,^^21^^)^ However, in healthy elderly individuals, these impairments are usually compensated for and generally do not promote harm to swallowing safety and overall health.^(^^5^^,^^12^^)^

The elderly survivor of laryngeal cancer treatment presents with dysphagia from an association of factors. The dry mouth is often cited as a risk factor for dysphagia arising from age. However, age-related physiological changes in saliva production are small and can be enhanced by the anticholinergic side effects of medications and radiation therapy.^(^^5^^,^^7^^,^^15^^,^^20^^,^^22^^)^

The reduction of oropharyngeal musculature volume is already known as a factor related to the swallowing alterations originated from aging. Feng et al.,^(^^23^^)^ demonstrated that the volume of the genius-hyoid muscle was significantly reduced in elderly individuals compared to the young. Moreover, they described that its volume was significantly reduced in elderly patients who aspired compared to those who did not.^(^^22^^)^ Butler et al.,^(^^24^^)^ demonstrated that the strength of the tongue was also significantly associated with aspiration status in older individuals.^(^^23^^)^

Just as aging, SCL promotes structural and therefore functional changes, which result in *deficits* in the feeding process. The surgical technique described by Laccourreye et al., in 1990, includes myotomy of the inferior pharyngeal constrictor muscle. Therefore, the region of the new hypopharynx presents less mucosal support by the remaining structure, which creates a food retention area in the pharyngoesophageal transition and pyriform recesses.^(^^8^^–^^10^^)^ The structural modification after resection and pexis in SCL CHEP promotes impact on the food bolus ejection and, consequently, on pharyngeal contraction sometimes insufficient and not modulated to its volume and viscosity, favoring the formation of residues in the posterior pharyngeal wall. In addition, the tongue base changes are added to the difficulties of epiglottis eversion, due to its surgical fixation, favoring the formation of residues and the penetration after swallowing. This procedure includes myotomy of the inferior pharyngeal constrictor muscle, a structure that, with the anterior laryngeal movement, should traction and allow the relaxation of the superior esophageal sphincter.^(^^8^^–^^11^^,^^16^^–^^18^^,^^22^^)^ This compromise makes the sphincter opening more difficult (lower amplitude and duration of opening) and favors the formation of residues and laryngotracheal aspiration.^(^^19^^)^

The health and quality of life of the elderly are influenced by multiple factors: social, physical, psychological, and cultural. Evaluating and promoting the health of the elderly mean considering variables from different fields of knowledge in a transdisciplinary action.^(^^12^^)^ Assistance to the elderly in swallowing must ensure the maintenance of quality of life, considering the losses arising from aging and the various possibilities coming from other comorbidities.^(^^5^^–^^7^^,^^12^^,^^20^^)^

Concomitant alcohol consumption and smoking were observed in most of the individuals in this study. Its synergistic effect is described in literature as being a risk factor associated with the increased occurrence of head and neck cancers.^(^^10^^,^^11^^,^^17^^)^ It is possible to understand that the oncologic patient presents an inferior aging quality than the others, due to several inadequate and unhealthy habits.^(^^2^^)^ The prevalence of elderly (80%) men (98.6%) is coherent with the profile of the population submitted to this procedure worldwide.^(^^16^^–^^19^^)^ A recent Brazilian study stated the need for attention to men’s health due to their less focus on health, devaluation of symptoms, abuse of toxic substances, and worse eating habits.^(^^17^^)^

The study presented limitations. No differential analysis of swallowing performance was performed with stratification of different consistencies and volumes. It is possible to infer that these aspects also influence the swallowing of patients submitted to SCL and elderly. A higher prevalence of penetration and greater probability of laryngotracheal aspiration in the elderly were observed, but it is necessary to understand that functional results and survival are multifactorial aspects. The number of arytenoids, individual anatomical variations, and the process of healing and adaptation of remaining structures may also interfere with the dynamics of swallowing and the interaction among its phases. Many of these aspects are difficult to control clinically and to measure, and were not included in the objectives of the work.

Due to the major involvement in the swallowing dynamics due to the surgical procedure, patients submitted to SCL CHEP have a greater probability of presenting food residues in different anatomical structures after swallowing. This aspect favors the presence of penetration and pulmonary aspiration even in individuals with no complaints. Age demonstrated to be a risk factor for pulmonary aspiration in the group. Speech therapy, through its various strategies, is essential at all times of treatment to ensure better functional results. Assertive speech therapy can help maintain a safe oral intake, managing the surgical and aging damage, and potentially increasing survival with higher quality.^(^^19^^,^^22^^)^

Supracricoid laryngectomy CHEP is a procedure with good oncologic control^(^^8^^,^^10^^,^^17^^,^^18^^)^ and, thus, patients are expected to have a long survival period. Our results alert to the need for long-term management adjustments and raise questions about a definitive discharge, the need for functional maintenance therapy, and periodic evaluation tests.

## CONCLUSION

In patients with no swallowing complaint in the late postoperative period of supracricoid laryngectomy, the prevalence of penetration, and the probability of laryngotracheal aspiration are higher in elderly people aged over 60 years than in individuals under 60 years. The possibility of interference of aging in functional results of oral ingestion of patients submitted to partial laryngectomy was observed in the safety and efficiency parameters.
