# Supplementary material for: Supracricoid laryngectomy: the impact of senescence on swallowing safety
Source: Einstein (Sao Paulo). 2021 Apr 28;19:eAO5715. doi: 10.31744/einstein_journal/2021AO5715 (PMC8081437; doi:10.31744/einstein_journal/2021AO5715)
Supplement: Translation

# Translation: Laringectomia supracricóidea: o impacto da senescência na segurança da deglutição

**Authors:** Andressa Silva de Freitas, Guilherme Maia Zica, Ana Catarina Alves e Silva, Fernando Luiz Dias, Emilson Queiroz Freitas, Izabella Costa Santos

**Affiliations:** 1 Rio de JaneiroRJ Brasil Instituto Nacional de Câncer José Alencar Gomes da Silva - INCA, Rio de Janeiro, RJ, Brasil.; 2 Rio de JaneiroRJ Brasil Fundação Oswaldo Cruz, Rio de Janeiro, RJ, Brasil.

**Keywords:** Laringectomia, Reabilitação, Deglutição, Envelhecimento, Transtornos de deglutição

## Abstract

**Objetivo:**

Investigar a associação entre o envelhecimento e os aspectos funcionais da deglutição (penetração laríngea e aspiração laringotraqueal) em indivíduos submetidos à laringectomia supracricóidea no período tardio e sem queixas.

**Métodos:**

Setenta pacientes, sendo 56 (80%) >60 anos e 14 (20%) <60 anos, em acompanhamento ambulatorial, após tratamento oncológico e sem queixas de deglutição, realizaram avaliação funcional por meio da videofluoroscopia da deglutição. A classificação das imagens foi realizada por meio da escala de penetração-aspiração desenvolvida por Rosenbek. O teste do *χ*^2^ e a regressão logística foram aplicados para associação das categorias de idade aos desfechos (penetração e aspiração).

**Resultados:**

Os pacientes com idade acima de 60 anos apresentaram maior prevalência de penetração (24,29%) e aspiração (48,57%) do que aqueles com idade inferior a 60 anos. Nesta amostra, a aspiração se mostrou associada à idade. Pacientes acima de 60 anos tiveram chance 27% maior de penetração durante a deglutição do que os com menos de 60 anos. Pacientes acima de 60 anos tiveram chance aproximadamente quatro vezes maior de aspiração laringotraqueal do que pacientes com menos de 60 anos.

**Conclusão:**

Em pacientes sem queixas de deglutição no pós-operatório tardio de laringectomia supracricóidea, há maior chance de aspiração laringotraqueal em idosos acima de 60 anos do que em indivíduos abaixo de 60 anos.

## INTRODUÇÃO

O envelhecimento é um processo natural que resulta em mudanças graduais e inevitáveis relacionadas à idade. Além de desencadear o desgaste orgânico e funcional, promove alterações nos aspectos emocionais, sociais e culturais.^(^^1^^)^ O envelhecer normal está ligado à capacidade de adaptação do indivíduo aos rigores e às agressões do meio ambiente. Dessa forma, cada sujeito envelhece a seu modo, dependendo de variáveis diversas, como sexo, origem, ambiente familiar, trabalho, ocupações e experiências vivenciadas.^(^^1^^–^^3^^)^ Para a Organização Mundial da Saúde (OMS), o limite de idade entre o indivíduo adulto e o idoso é 65 anos em nações desenvolvidas e 60 anos nos países emergentes.^(^^4^^)^

A exposição ao estresse ou ao tabagismo, a falta de exercícios ou a nutrição inadequada são fatores que contribuem para determinar a qualidade do envelhecimento do indivíduo.^(^^2^^)^ A senescência e a doença não podem ser tratados como fatores intimamente dependentes ou interligados, porém é possível compreender que o idoso apresenta maior vulnerabilidade ao adoecer.^(^^3^^)^

A disfagia é uma disfunção da deglutição, definida pela dificuldade ou incapacidade de mover efetivamente o bolo alimentar da boca para o estômago. Esse distúrbio pode afetar até 13% da população com 65 anos ou mais e 51% dos idosos institucionalizados.^(^^5^^)^ A deglutição pode ser impactada com aumento da idade, acúmulo de comorbidades e hospitalização. Existem diversas doenças com potencial para desencadear disfagia, como acidente vascular cerebral e tumores de cabeça e pescoço. Nesses grupos, estudos sugerem que sua prevalência aumenta com a sobrevida dos indivíduos, o que pode significar que as mudanças do envelhecimento contribuem para o agravamento da disfagia.^(^^6^^,^^7^^)^

A laringectomia supracricóidea (LSC) é um procedimento cirúrgico parcial horizontal indicado para os tumores T1b a T4a da região glótica e supraglótica, cujas principais vantagens são a preservação parcial de funções laríngeas e a ausência de traqueostomia permanente.^(^^8^^)^ Sua técnica consiste na remoção do pedículo da epiglote, cartilagem tireoidiana, ventrículos la ríngeos, pregas vocais, pregas vestibulares e espaço pa raglótico. Permanece uma ou ambas unidades cricoaritenóideas, a epiglote e a cricoide. Sua reconstrução cirúrgica ocorre, entre variações, por meio da cricohioidoepiglotopexia (CHEP), na qual é realizada uma pexia (sutura) entre a cartilagem cricoide, a epiglote e o osso hioide, que eleva o complexo laríngeo ao nível do osso hioide.^(^^8^^,^^9^^)^ A LSC promove alterações na biodinâmica da deglutição devido à perda de importantes estruturas de pro teção das vias aéreas inferiores.^(^^10^^,^^11^^)^

A atrofia da musculatura faríngea tem sido identificada como um processo biológico natural do envelhecimento, no qual observa-se um aumento na área e no volume da faringe em indivíduos saudáveis. Sabe-se que idosos saudáveis com cavidades faríngeas maiores apresentam piora da constrição faríngea e, consequentemente, maior quantidade de resíduos em recessos faríngeos e valécula.^(^^5^^)^ O envelhecimento natural promove a calcificação das cartilagens laríngeas, menor mobilidade muscular e cicatrização/ vascularização débil no pós-operatório, o que é negativamente relacionado às questões cirúrgicas.^(^^7^^,^^9^^,^^11^^)^ Acredita-se que o envelheci mento possa potencializar os resultados oncológicos negativos das laringectomias por diferentes fatores. Esse aspecto pode comprometer paulatinamente o processo de deglutição, devido à somatória da presbiafagia e do rearranjo anatomofisiológico da neoglote.

## OBJETIVO

Investigar a associação entre o envelhecimento e os aspectos funcionais da deglutição em indivíduos submetidos à laringectomia supracricóidea no período tardio.

## MÉTODOS

Estudo seccional realizado na Seção de Cirurgia de Câncer de Cabeça e Pescoço do Instituto Nacional de Câncer do Brasil (INCA), Rio de Janeiro (RJ), Brasil. A aprovação para este estudo foi obtida do Comitê de Ensino e Pesquisa da instituição, sob parecer 3.892.889, CAAE: 26331314.2.0000.5274.

As diferentes idades para definição do idoso segundo a OMS existem por conta das menores taxas de sobrevida e de questões associadas à saúde pública.^(^^4^^,^^12^^)^ A idade, neste estudo, foi considerada no momento da avaliação, e não da operação. Setenta pacientes, sendo 56 (80%) >60 anos e 14 (20%) <60 anos, em acompanhamento ambulatorial, após tratamento oncológico e sem queixas de deglutição, realizaram avaliação funcional por meio da videofluoroscopia da deglutição (VFD). A classificação das imagens foi realizada por meio da Escala de Penetração e Aspiração desenvolvida por Rosenbek et al., em 1996, e utilizada como parâmetro para analisar a presença e a ausência de penetração laríngea e aspiração laringotraqueal.^(^^13^^)^

Como critério de inclusão, os pacientes deveriam ser maiores de 18 anos e terem sido tratados cirurgicamente pela técnica descrita por Majer et al.,^(^^8^^)^ e aprimorada por Laccourreye et al.,^(^^9^^)^ sem doença ativa no momento e com pelo menos 6 meses de tratamento oncológico, sem recorrência local ou metástases à distância. Os pacientes apresentavam-se sem nenhuma queixa de deglutição, de alta do serviço de fonoaudiologia, sem traqueostomia ou sonda de alimentação. A radioterapia adjuvante como parte do protocolo de tratamento não foi um critério de exclusão. Foram excluídos pacientes com comprometimentos cognitivos e/ou linguístico e aqueles que realizaram outro tipo de intervenção cirúrgica na região da cabeça e pescoço antes ou após a LSC CHEP.

Os exames videofluoroscópicos foram realizados no Departamento de Radiologia do INCA, de acordo com o protocolo baseado em Logemann^(^^14^^)^ e adaptado no Laboratório Interdisciplinar de Cabeça e Pescoço (LICEP). Foi utilizada uma máquina de raios-X remote-controlled Siemens Axiom Iconos MD, número de série 13020. Todos os segmentos de vídeo foram gravados em um plano lateral, com taxa de captura de imagem de 30 quadros por segundo, e arquivados em *Picture Archiving and Communication Systems* (PACS), permitindo armazenar documentos para revisão e análise posteriores.

O protocolo de preparação das consistências foi o contraste oferecido em um copo, usando diluições de sulfato de bário (SB) 100% Bariogel^®^, água mineral e espessante alimentar Resource^®^ ThickenUp Clear. Foram ofertadas quatro consistências: líquida 5mL (2,5mL de água + 2,5mL de SB), 10mL (5mL de água + 5mL de SB) e 20mL (10mL de água + 10mL de SB); semilíquida 5mL de SB, 10mL de SB e 20mL de SB; pastosa 5mL (5mL de SB + 1,2g de espessante), 10mL (10mL de SB + 2,4g de espessante) e 20mL (20mL de SB + 3,6g de espessante); sólida (biscoito umedecido em SB). Durante o exame, todos os sujeitos foram posicionados em vista lateral, o mais próximo possível da mesa e do intensificador, visando evitar distorções na imagem fluoroscópica.

Este é um estudo com análise descritiva e regressão estatística simples. Teste do *χ*^2^ e regressão logística foram aplicados para associação das categorias de idade aos desfechos (penetração e aspiração), utilizando o programa R versão 3.5.3.

## RESULTADOS

Foram avaliados 70 pacientes, predominantemente idosos, com idade superior a 60 anos (80%), do sexo masculino (98,57%), casados (75,71%) e com baixa escolaridade (57,14%). Mais de 80% dos indivíduos eram tabagistas e pelo menos 60% referiram etilismo no momento do diagnóstico. Somando-se as categorias solteiro, divorciado e viúvo, foram obtidos 24,28% (n=17) que não relataram companheiro no momento da coleta. Quanto ao estadiamento, observaram-se proporções de 38,57% e 32,86% entre indivíduos de estadiamento II e III (Tabela 1).

**Tabela 1:** Distribuição das informações sobre hábitos, variáveis clínicas e informações sociodemográficas

Variáveis		
Sexo		
	Masculino	69 (98,6)
	Feminino	1 (1,4)
Idade em julho de 2017, anos		
	<60	14 (20)
	≥60	56 (80)
Estado conjugal		
	Casado	53 (75,7)
	Solteiro	8 (11,4)
	Divorciado	3 (4,3)
	Viúvo	6 (8,6)
Escolaridade		
	Analfabeto	2 (2,9)
	Ensino fundamental	48 (54,3)
	Ensino médio	25 (35,7)
	Ensino superior	5 (7,1)
Tabagista ao diagnóstico		57 (81,4)
Etilista ao diagnóstico		46 (65,7)
Estadiamento clínico		
	I	19 (27,1)
	II	27 (38,6)
	III	23 (32,9)
	IV	1 (1,4)
Sonda nasogástrica, dias		
	≤45	38 (54,3)
	46-89	20 (28,6)
	>90	12 (17,1)
Traqueostomia, dias		
	≤45	46 (65,7)
	46-89	12 (17,1)
	>90	12 (17,1)
Radioterapia		22 (31,9)
Aritenoides preservas		
	1	32 (47,7)
	2	38 (54,3)

Resultados expressos por n (%).

As retiradas de sonda nasogástrica e traqueostomia ocorreram até 45 dias em 54,28% e 65,71%, respectivamente. A radioterapia foi realizada em 31,9% dos pacientes (Tabela 1).

Os pacientes com idade acima de 60 anos apresen taram maior prevalência de penetração (24,29%) e aspiração (48,57%) do que os com idade inferior a 60 anos. Nesta amostra, a aspiração se mostrou associada à idade (Tabela 2).

**Tabela 2:** Distribuição do grupo por idade, segundo prevalência de penetração e aspiração laríngea

Idade, anos	Penetração			Aspiração		
	Sim	Não	Valor de p	Sim	Não	Valor de p
<60	5 (7,14)	9 (12,86)	0,787	12 (17,14)	2 (2,86)	
≥60	17 (24,29)	39 (55,71)		34 (48,57)	22 (31,43)	0,063

Resultados expressos por n (%).

Pacientes acima de 60 anos tiveram chance 27% maior de penetração durante a deglutição do que os com menos de 60 anos. Também apresentaram chance aproximadamente quatro vezes maior de aspiração laringotraqueal do que pacientes com menos de 60 anos (Tabela 3).

**Tabela 3:** Regressão simples do grupo por idade, segundo prevalência de penetração e aspiração laríngea

Idade, anos	Penetração			Aspiração		
	RC	IC95%	Valor de p	RC	IC95%	Valor de p
<60	1			1		
≥60	1,27	0,35-4,28	0,700	3,88	0,94-26,51	0,094

RC: razão de chances; IC95%: intervalo de confiança de 95%.

## DISCUSSÃO

A motivação deste estudo baseou-se na necessidade de compreender a correlação entre a presbifagia e a segurança e eficácia de deglutição em pacientes submetidos à LSC CHEP. A técnica em questão visa à manutenção parcial das funções de deglutição e fonação como alternativa a tratamentos com maior potencial para provocar alterações funcionais, como a radioterapia exclusiva e a laringectomia total, para o caso de tumores avançados.^(^^8^^,^^9^^,^^15^^)^ Os dados encontrados neste estudo sugerem maior chance de aspiração laringotraqueal em indivíduos acima de 60 anos submetidos à LSC CHEP em comparação aos com menos de 60 anos submetidos ao mesmo procedimento, ambos sem queixas de deglutição ou limitações de via oral.

A LSC CHEP é um procedimento cirúrgico prescrito tradicionalmente para o tratamento de tumores de estágios intermediário e avançado, o que justifica a prevalência de indivíduos com estadiamento clínico dois e três (71,5%).^(^^9^^,^^11^^,^^16^^–^^18^^)^ O procedimento demonstrou ser uma alternativa viável à laringectomia total, com preservação parcial das funções laríngeas em todos os indivíduos.

A aspiração traqueal é um desfecho clínico crônico conhecido da LSC, com incidências aproximadas de 40% no pós-operatório, mesmo em indivíduos sem queixas.^(^^17^^–^^19^^)^ Os *deficits* no fechamento glótico e proteção das vias aéreas, devido à retirada de aproximadamente 70% do órgão, e as alterações de sensibilidade na neolaringe podem justificar parcialmente tais achados.^(^^19^^)^

Os pacientes com idade acima de 60 anos apresentaram maior prevalência de penetração e aspiração durante a análise objetiva da deglutição. Diversos fatores na presbifagia podem influenciar no resultado funcional dos indivíduos idosos, como perda de peso e massa muscular, redução da elasticidade dos tecidos, alterações na coluna cervical, redução da produção de saliva, comprometimento do estado dentário e saúde oral, sensibilidade oral e faríngea reduzidas, função olfativa e gustativa reduzidas e capacidade cerebral compensatória reduzida.^(^^5^^,^^6^^,^^12^^,^^20^^)^ Evidentemente, todos esses aspectos aumentam a suscetibilidade à disfagia e se sobrepõem aos comprometimentos cirúrgicos do tratamento oncológico.

Acredita-se que as alterações oriundas do envelhecimento podem afetar todas as fases da deglutição independente da associação com outras comorbidades. Fase oral prolongada, pressão reduzida da língua, atraso no disparo do reflexo da deglutição, atraso no fechamento da laringe, diminuição do volume da deglutição e aumento de resíduos e taxa de penetração são descritos como alterações típicas de pessoas com idade avançada.^(^^20^^,^^21^^)^ Porém, em indivíduos idosos saudáveis, esses comprometimentos normalmente são compensados e, geralmente, não promovem prejuízos à segurança da deglutição e saúde geral.^(^^5^^,^^12^^)^

O idoso sobrevivente do tratamento de um câncer de laringe apresenta disfagia oriunda de uma associação de fatores. A boca seca é frequentemente citada como um fator de risco de disfagia proveniente da idade. Entretanto, as alterações fisiológicas relacionadas à idade na produção de saliva são pequenas e podem ser potencializadas pelos efeitos colaterais anticolinérgicos de medicações e da radioterapia.^(^^5^^,^^7^^,^^15^^,^^20^^,^^22^^)^

A redução do volume da musculatura orofaríngea já é conhecida como fator relacionado às alterações da deglutição oriundas do envelhecimento. Feng et al.,^(^^23^^)^ demonstraram que o volume do músculo gênio hióideo foi significativamente reduzido em indivíduos idosos em comparação aos jovens. Além disso, descrevem que seu volume foi significativamente reduzido em pacientes idosos que aspiram em comparação com os que não aspiram.^(^^22^^)^ Butler et al.,^(^^24^^)^ demonstraram que a força da língua também estava significativamente associada com *status* de aspiração em indivíduos mais velhos.^(^^23^^)^

Assim como o envelhecimento, a LSC promove al te rações estruturais e, por conseguinte, funcionais, que resultam em *deficits* no processo de alimentação. A técnica cirúrgica descrita por Laccourreye et al., em 1990, inclui a miotomia do músculo constritor inferior da faringe. Portanto, a região da nova hipofaringe apresenta menor sustentação mucosa pela estrutura remanescente, que cria uma área de retenção de alimento na transição faringoesofágica e recessos piriformes.^(^^8^^–^^10^^)^ A modificação estrutural após a ressecção e pexia na LSC CHEP promove impacto na ejeção do bolo alimentar e, consequentemente, a contração faríngea por vezes insuficiente e não modulada ao seu volume e viscosidade, favorecendo a formação de resíduos em parede posterior de faringe. Além disso, as alterações de base de língua se somatizam às dificuldades de eversão da epiglote, devido à sua fixação cirúrgica, favorecendo a formação de resíduos e a presença de penetração após a deglutição. Esse procedimento inclui a miotomia do músculo constrictor inferior da faringe, estrutura que, com o movimento anterior da laringe, deveria tracionar e permitir o relaxamento do esfíncter esofágico superior.^(^^8^^–^^11^^,^^16^^–^^18^^,^^22^^)^ Esse comprometimento dificulta a abertura do esfíncter (menor amplitude e duração de abertura) e favorece a formação de resíduos e aspiração laringotraqueal.^(^^19^^)^

A saúde e a qualidade de vida dos idosos sofrem influência de múltiplos fatores: sociais, físicos, psicológicos e culturais. Avaliar e promover a saúde do idoso significam considerar variáveis de distintos campos do saber em uma atuação transdisciplinar.^(^^12^^)^ A assistência em deglutição ao idoso deve garantir a manutenção da qualidade de vida, considerando as perdas advindas do envelhecimento e as diversas possibilidades provenientes de outras comorbidades.^(^^5^^–^^7^^,^^12^^,^^20^^)^

O consumo de álcool e o tabagismo concomitantes foram observados em grande parte dos indivíduos deste estudo. Seu efeito sinérgico é descrito na literatura, sendo fator de risco associado ao aumento da ocorrência dos cânceres de cabeça e pescoço.^(^^10^^,^^11^^,^^17^^)^ É possível compreender que o paciente oncológico apresenta qualidade do envelhecimento inferior a dos demais, devido aos diversos hábitos inadequados e não saudáveis.^(^^2^^)^ A prevalência de homens (98,6%) idosos (80%) é coerente com o perfil da população submetida a esse procedimento no mundo.^(^^16^^–^^19^^)^ Estudo nacional recente afirma a necessidade da atenção à saúde do homem devido a seu menor cuidado com a saúde, desvalorização de sintomas, abuso de substâncias tóxicas e piores hábitos alimentares.^(^^17^^)^

O estudo apresentou limitações. Não foi realizada análise diferencial do desempenho da deglutição com estratificação de diferentes consistências e volumes. É possível inferir que esses aspectos também influenciam na deglutição do paciente submetido à LSC e idoso. Observaram-se maior prevalência de penetração e maior chance de aspiração laringotraqueal em idosos, porém é necessário compreender que os resultados funcionais e a sobrevida são aspectos multifatoriais. É possível que o número de aritenoides, as variações anatômicas individuais e o processo de cicatrização e de adaptação das estruturas remanescentes possam também interferir na dinâmica da deglutição e na interação entre suas fases. Muitos destes aspectos são de difícil controle clínico e mensuração e, além disso, não fizeram parte do objetivo do trabalho.

Devido aos grandes comprometimentos na dinâmica da deglutição provenientes do procedimento cirúrgico, pacientes submetidos à LSC CHEP apresentam maior chance de apresentar resíduos alimentares em diferentes estruturas anatômicas após a deglutição. Este aspecto favorece a presença de penetração e broncoas piração mesmo em indivíduos sem queixas. A idade demonstrou-se aspecto de risco para broncoaspiração no grupo. A fonoaudiologia, por meio de suas diversas estratégias, é essencial em todos os momentos do tratamento para assegurar melhores resultados funcionais. Uma fonoterapia assertiva pode auxiliar na manutenção de uma ingesta via oral segura, gerenciando os danos cirúrgicos e oriundos do envelhecimento e aumentando potencialmente a sobrevida com maior qualidade.^(^^19^^,^^22^^)^

A LSC CHEP é um procedimento com bom controle oncológico^(^^8^^,^^10^^,^^17^^,^^18^^)^ e, sendo assim, é esperado que os pacientes tenham um período longo de sobrevida. Nossos resultados alertam para a necessidade no ajuste de condutas a longo prazo e levantam questionamentos sobre alta definitiva, necessidade de terapia de manutenção funcional e exames de avaliação de forma periódica.

## CONCLUSÃO

Em pacientes sem queixas de deglutição no pós-operatório tardio de laringectomia supracricóidea, a prevalência de penetração e a chance de aspiração laringotraqueal são maiores em idosos acima de 60 anos do que em indivíduos abaixo de 60 anos. Observou-se a possibilidade da interferência do envelhecimento nos resultados funcionais da ingesta oral de pacientes submetidos à laringectomia parcial nos parâmetros de segurança e eficiência.
